# The necroptosis-related signature and tumor microenvironment immune characteristics associated with clinical prognosis and drug sensitivity analysis in stomach adenocarcinoma

**DOI:** 10.18632/aging.205690

**Published:** 2024-03-27

**Authors:** Biao Yang, Yingnan Wang, Tao Liu, Meijing Zhang, Tianhang Luo

**Affiliations:** 1 Department of General Surgery, Changhai Hospital, Naval Medical University, Shanghai 200433, China; 2 Henan University of Science and Technology, Henan 471000, China; 3 Department of Emergency, Changhai Hospital, Naval Medical University, Shanghai 200433, China; 4 Department of Oncology, Changhai Hospital, Naval Medical University, Shanghai 200433, China

**Keywords:** necroptosis, stomach adenocarcinoma, prognostic signature, immune infiltration, drug sensitivities

## Abstract

Purpose: Necroptosis plays an important role in the tumorigenesis, development, metastasis, and drug resistance of malignant tumors. This study explored the new model for assessing stomach adenocarcinoma (STAD) prognosis and immunotherapy by combining long noncoding RNAs associated with necroptosis.

Methods: Patient clinical data and STAD gene expression profiles were curated from The Cancer Genome Atlas (TCGA). Immune-related genes were sourced from a specialized molecular database. Perl software and R software were used for data processing and analysis. Necroptosis-related lncRNAs in STAD were pinpointed via R’s correlation algorithms. These lncRNAs, in conjunction with clinical data, informed the construction of a prognostic lncRNA-associated risk score model using univariate and multivariate Cox regression analyses. The model’s prognostic capacity was evaluated by Kaplan-Meier survival curves and validated as an independent prognostic variable. Further, a nomogram incorporating this model with clinical parameters was developed, offering refined individual survival predictions. Subsequent analyses of immune infiltration and chemosensitivity within necroptosis-related lncRNA clusters utilized an arsenal of bioinformatic tools, culminating in RT-PCR validation of lncRNA expression.

Results: Through rigorous Cox regression, 21 lncRNAs were implicated in the risk score model. Stratification by median risk scores delineated patients into high- and low-risk cohorts, with the latter demonstrating superior prognostic outcomes. The risk model was corroborated as an independent prognostic indicator for STAD. The integrative nomogram displayed high concordance between predicted and observed survival rates, as evidenced by calibration curves. Differential immune infiltration in risk-defined groups was illuminated by the single sample GSEA (ssGSEA), indicating pronounced immune presence in higher-risk patients. Tumor microenvironment (TME) analysis showed that cluster-C3 had the highest score in the analysis of the three TMEs. Through the differential analysis of immune checkpoints, it was found that almost all immune checkpoint-related genes were expressed differently in various tumor clusters. Among them, CD44 expression was the highest. By comparing all drug sensitivities, we screened out 29 drugs with differences in drug sensitivity across different clusters. Risk score gene expression identification results showed that these lncRNAs were abnormally expressed in gastric cancer cell lines.

Conclusions: This investigation provides a robust methodological advance in prognosticating and personalizing immunotherapy for STAD, leveraging quantitatively derived tumor cluster risk scores. It posits the use of necroptosis-related lncRNAs as pivotal molecular beacons for guiding therapeutic strategies and enhancing clinical outcomes in STAD.

## INTRODUCTION

Stomach adenocarcinoma (STAD) is a widespread malignancy that poses a significant global health challenge. According to the 2012 World Cancer Statistics, STAD accounted for 6.8% of all malignant tumors, with 951,000 new cases reported, ranking it fifth among malignancies [1]. Although surgical resection remains the primary treatment for advanced STAD, the 5-year survival rate after surgery remains low. Based on adjuvant therapy, the 5-year survival rate was approximately 20% to 25% [2]. In recent years, immunotherapy has emerged as one of the most promising strategies in cancer treatment, demonstrating remarkable therapeutic efficacy in tumors such as melanoma, non-small cell lung cancer, and kidney cancer [3–5]. Immunotherapy research on STAD has become a research hotspot, and various immunotherapy strategies have been developed, including immune checkpoint inhibitors, cellular adoptive immunotherapy, cancer vaccines, etc. [6]. These immunotherapy methods aim to increase the patient’s immune system response to tumors or the immunogenicity of the tumors [7]. However, it is important to note that many new immunotherapies are still in the early stages of clinical research. Hence, it was deemed of utmost importance to investigate the mechanism of immunotherapy in STAD, along with the immune, molecular, and genetic characteristics of STAD patients.

Apoptosis was the earliest discovery of programmed cell death, first proposed in 1972 by Kerr JF et al. [8]. In 2005, American scholars Degterev found that receptor-interacting protein kinase 3 (RIPK3) activates mixed lineage kinase domain-like protein (MLKL) leading to cell membrane rupture of the cell membrane in the form of cell necrosis, called necroptosis [9]. Subsequently, other forms of programmed cell death such as pyroptosis, ferroptosis, and autophagy, were discovered. Necroptosis has been found to have a regular regulatory mechanism, which is a necrotic form of cell death that occurs when the apoptosis pathway is inhibited. Further research has revealed that necroptosis is not only involved in the inflammatory pathological mechanism, but is also closely related to the occurrence and development of tumors, as well as the mechanism of drug resistance. Furthermore, it is worth noting that necroptosis may potentially contribute to the initiation of immunogenicity and facilitate natural anti-cancer immune surveillance [10–12]. Given the involvement of necroptosis in the pathogenesis of various diseases, it is imperative to investigate its role in the development of STAD and to devise novel therapeutic approaches for this condition.

Some studies have shown that the occurrence and development of STAD were accompanied by a variety of dysregulation of the long non-coding RNAs (lncRNAs) [13, 14]. The abnormal expression of lncRNAs can ultimately impact epigenetics, resulting in the development of malignant tumor phenotypes. Targeting the proliferation, infiltration, and metastasis of tumor cells through early intervention could potentially serve as a treatment option for STAD. Therefore, it can be argued that identifying the biological behavior of lncRNAs may provide a basis for the diagnosis and treatment of STAD. It has been suggested that lncRNAs have the ability to alter the activity of multiple signaling pathways by regulating target gene expression, such as Wnt/β-catenin [15, 16], PI3K-AKT/mTOR [17, 18], JAK/STAT [19], NF-κ B [20], thereby potentially promoting or inhibiting tumor cell activity. The role of lncRNA in tumors has only been preliminarily recognized in recent years, and further study is needed to understand the specific regulatory mechanism in STAD. Therefore, a comprehensive understanding of the lncRNAs regulatory network associated with necroptosis will help deepen the understanding of the mechanism of STAD and may provide new ideas and methods for the clinical immunotherapy of STAD.

The in-depth study of necroptosis has been found to deepen our understanding of the way cells die and has helped in the study of the development and variation of different disease models. Additionally, the relationship between necroptosis and the occurrence, development, and outcome of tumors is an area of ongoing research interest. The study of the role of necroptosis in tumor pathogenesis has the potential to advance the development of new therapeutic targets. Although there is currently limited research on necroptosis-related lncRNAs in STAD, this study aims to investigate their expression and prognostic significance in STAD. The findings will contribute to a better understanding of the pathogenesis of STAD and provide valuable insights for the development of drugs targeting related molecular pathways.

## MATERIALS AND METHODS

### Collection and processing of clinical data

RNA-seq data and clinical information were obtained from the TCGA database (https://portal.gdc.cancer.gov/). The dataset included 375 tumor samples and 32 normal samples. Perl software was utilized for data integration, including the extraction of necrotizing apoptosis gene expression data, lncRNA expression data, and corresponding clinical data. To ensure comparability of gene expression across samples, the data were converted into TPM values. This adjustment made the gene expression data more comparable between samples, increasing the similarity to the transcript samples produced by the microarray. The gene expression data extraction project included gene name, sample number, and expression value, while the clinical data included patient number, survival time, survival status, age, gender, and TNM staging.

### Selection of necroptosis-related genes and lncRNAs

According to studies related to necroptosis, a total of 67 genes related to necroptosis were identified. The expression of these genes was extracted using the ‘limma’ package in R (Supplementary Table 1). Abnormally expressed lncRNAs were screened using the criteria of | Log_2_ fold change (FC)|> 1 and p < 0.05. Necroptotic-related lncRNAs were identified by correlating necroptosis-related genes with abnormally expressed lncRNAs using R software.

### Establishment and verification of risk prognostic model

Prognostic models were established by examining the expression levels of necroptosis-associated lncRNAs and corresponding STAD clinical data. Initially, the relationship between the expression levels of necroptosis-related lncRNAs and overall survival (OS) was assessed using univariate Cox regression analysis. Subsequently, necroptosis-related lncRNAs that were significantly associated with STAD prognosis were identified based on a P-value of less than 0.01. Subsequently, the screened necroptosis-related lncRNAs were included in the multivariate Cox regression analysis. The necroptosis-related lncRNAs that ultimately constituted the riskScore model were selected based on the optimal Akaike information criterion (AIC) simulation criteria. The model formula is as follows: [21]:


$$\text{Risk score}=\sum_{i\;=\;1}^{N}\left(\text{Ei}*\text{Ci}\right)$$

The variable N represents the number of necroptosis-related lncRNAs used to construct the riskScore model. Ci denotes the coefficient of necroptosis-related lncRNAs, while Ei represents their expression level.

Using this risk scoring model, patients are assigned a corresponding risk score. Based on the median risk score, patients can be classified into either high-risk or low-risk groups. The Kaplan-Meier (K-M) method was utilized to analyze the survival of high- and low-risk groups, and the difference in total survival time was examined using the log-rank test. In order to further evaluate this model, risk scores and other clinical features were subjected to univariate and multifactor Cox regression analysis to determine whether they were independent prognostic factors.

### Construction of nomogram

The nomogram of STAD is constructed using variables such as age, gender, TNM staging, and risk score. To evaluate the consistency between actual and predicted survival, calibration curves are plotted. To locate the corresponding point on the nomogram, patient variables are used to find the corresponding axis. A vertical line is then drawn over this point, and the value of the point that intersects the score axis represents the fraction of the variable. The total score is calculated by summing the fractions for each variable. Similarly, the total score value was observed on the survival rate axis, representing the likelihood of the patient’s survival during the corresponding time period [22].

### Gene set enrichment analysis (GSEA)

GSEA 4.0.3 was used to analyze the effects of genome-wide expression changes on various biological functions and pathways in patients in the high and low-risk groups. GSEA was used as a reference “c5: gene ontology (GO) gene sets” (c5.all.v7.0.sym-bols.gmt) and “c2: curated gene sets” (c2.cp.kegg.v7.0.symbols.gmt) from the molecular signatures database (MSigDB). The number of permutations was set to repeat 1,000 times. P < 0.05 and false discovery rate (FDR) less than 0.05 gene set as significantly enriched gene sets.

### Immune-related and drug-sensitivity analysis

The TIMER2.0 tool was utilized to evaluate the potential correlation between the expression of necroptosis-lncRNAs and the abundance of tumor-infiltrating immune cells (TIIC), such as CD4+ T cells, CD8+ T cells, macrophages, and others. Furthermore, to determine the relative changes in gene expression between groups, the CIBERSORT deconvolution algorithm, which is based on gene expression, was employed [23]. The National Cancer Institute Genomic Data Commons was the source of information on the immune-related characteristics of STAD samples. The 375 tumor samples were divided into high- and low-risk groups using the median risk score as the cut-off value. This was done to analyze and assess the effect of necroptosis-related lncRNAs on the immune system microenvironment. The R package ‘ggpubr’ was used to analyze differences in tumor microenvironment (TME) scores and immune checkpoints between the low- and high-risk groups.

The gene set variation analysis (GSVA) is a non-parametric unsupervised analysis method that can be used to assess gene collection enrichment in the transcriptome. By comprehensively scoring the gene set of interest, GSVA can determine the biological function of the sample, turning the gene level change into a pathway level change. The study utilized the Molecular Signatures Database (v7.0) (http://software.broad-institute.org/gsea/msigdb) to obtain the gene set, which was then scored using the GSVA algorithm to evaluate potential biological functional changes across various samples.

For the calculation of the TME score, there were currently methods of mean-weighted coefficient GSEA and principal component analysis (PCA). In order to provide a comprehensive assessment of the various scores, we integrated the three most commonly used methods for single-sample gene collection enrichment analysis: GSEA, principal component analysis PCA, and T-distributed stochastic neighbor embedding (t-SNE). Additionally, the K-M method was used to plot the survival curves of each group.

The ‘ConsensusClusterPlus’ package was used to perform the K-Medoid with cluster values ranging from 2 to 9, and the k value with higher cluster stability was selected based on the clustering effect [24]. The Genomics of Drug Sensitivity in Cancer (GDSC) used the half-maximal inhibitory concentration (IC_50_) to detect drug reactions. Tumor typing was performed using immunoassays, and drug sensitivity testing was conducted using the ‘ggpubr’ package in R software.

### Cell lines and culture

The human GC cell lines SGC-7901, MGC-803, and MKN-45, as well as the human normal gastric epithelial cell line GES-1, were obtained from the Institute of Biochemistry and Cell Biology of the Chinese Academy of Sciences in Shanghai, People’s Republic of China. They were cultured in Roswell Park Memorial Institute (RPMI) 1640 supplemented with 10% heat-inactivated fetal bovine serum (FBS), 100 U/mL of penicillin, and 100μg/mL streptomycin sulfate, in a humidified atmosphere containing 5% CO_2_ at 37° C.

### RNA preparation and quantitative real-time PCR

Total RNA was extracted from tissues or cultured cells using TRIzol reagent (Invitrogen, Carlsbad, CA, USA). For qRT-PCR, 1 μg RNA was reverse transcribed into cDNA with Reverse Transcription Kit (Takara, Dalian, China). Real-time PCR was performed with SYBR Premix ExTaq II Kit (Takara, Dalian China). The sequence of primers used in the detection is shown in Supplementary Table 2. The qRT-PCR assays and data collection were performed on ABI 7500, and relative expression was assessed by the 2^−∆Ct^ method, and converted to fold changes using the 2^−ΔΔCt^ method.

### Statistical analysis

R 3.6.2 Software was used for statistical analysis. The survival receiver operating characteristic (ROC) area under the curve (AUC) was calculated through the R software package, and clinical parameter differences were examined using an independent t-test. Qualitative data were compared using the Kruskal-Wallis H test. Differences in the proportion of infiltrated immune cells in the samples were analyzed using the log-rank test. The Pearson analyzed the correlation between the degrees of infiltration of various immune cells. P < 0.05 was statistically significant.

### Availability of data and materials

All data generated or analyzed during this study are included in this published article.

## RESULTS

### Necroptosis-related lncRNAs in STAD

**Figure 1:**
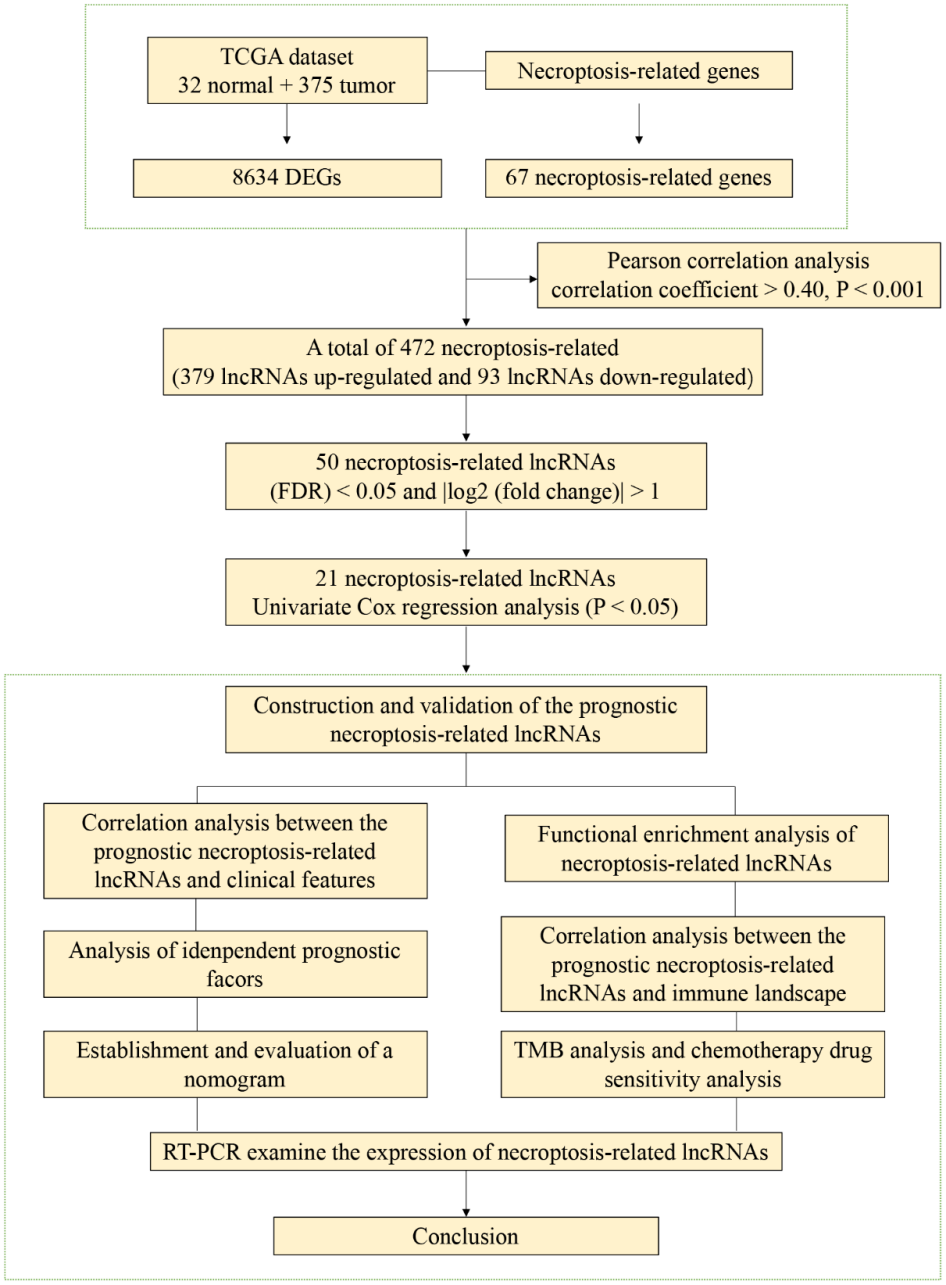
The flowchart of this study.

**Figure 2:**
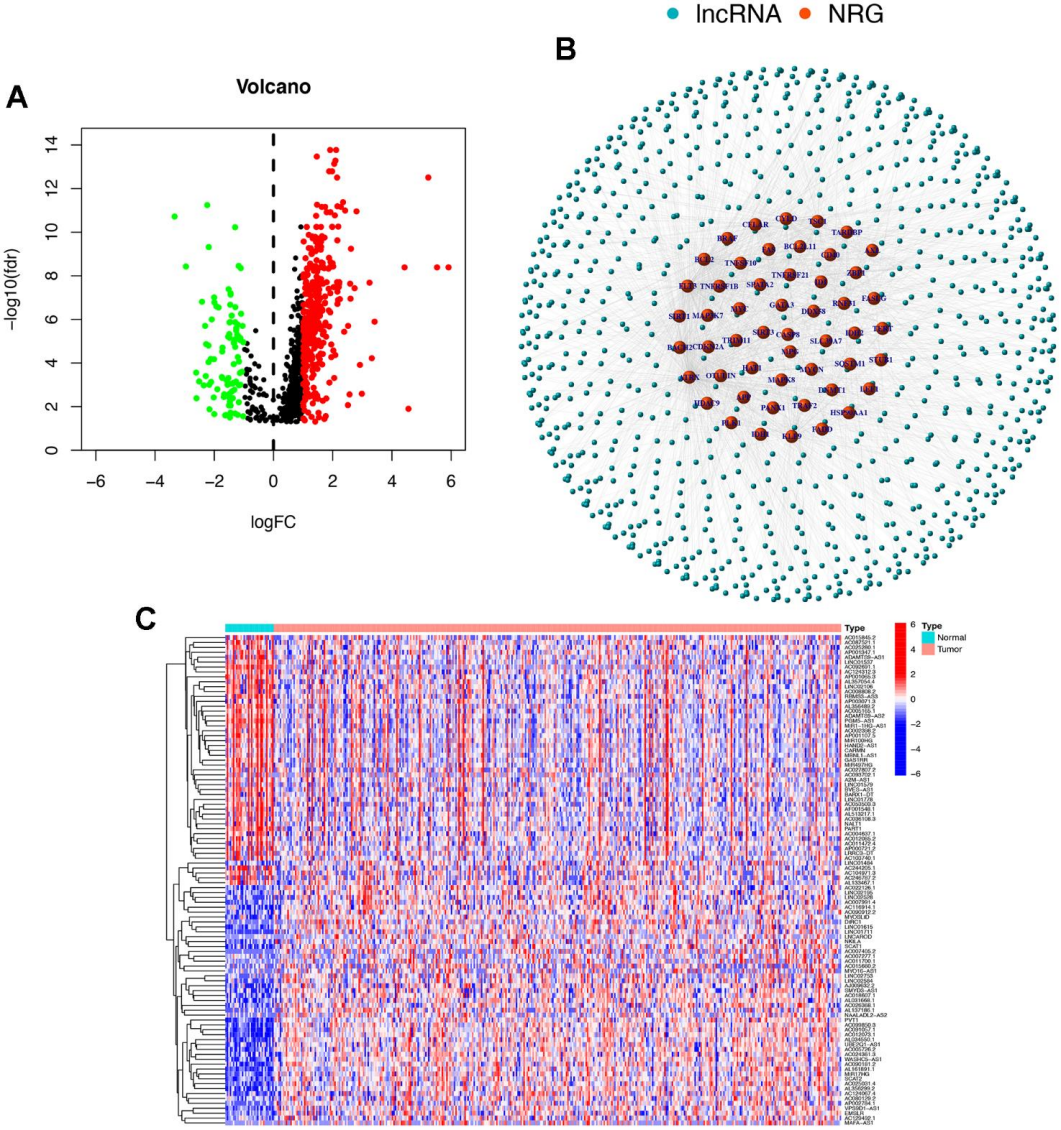
**Necroptosis-related lncRNAs in STAD.** (**A**) The volcano plot of all lncRNAs differential expressions associated with necroptosis in STAD. The red represents the expression of upregulation, the green represents the expression of downregulation, and the black represents that the expression has not changed. (**B**) The analytical relationship network diagram between necroptosis-related genes and lncRNA. (**C**) The heat maps were plotted based on the differential gene expression of the first 50 lncRNAs with the most significant upregulation and downregulation. FC, fold change; NRG, necroptosis gene.

The study process is shown in Figure 1. Gene expression data from 375 tumour samples and 32 normal samples of STAD patients were obtained from TCGA. In Figure 2A, all lncRNAs with differential expressions associated with necroptosis in STAD are displayed. A total of 472 abnormally expressed lncRNAs were analysed based on the expression levels of 67 necroptosis-related genes, of which 379 lncRNAs were up-regulated and 93 lncRNAs were down-regulated. In Figure 2B, an analytical relationship network diagram is displayed between necroptosis-related genes and lncRNA. The heat maps were plotted based on the differential gene expression of the first 50 lncRNAs with the most significant upregulation and downregulation (Figure 2C).

### Construct the risk prognostic model

**Figure 3:**
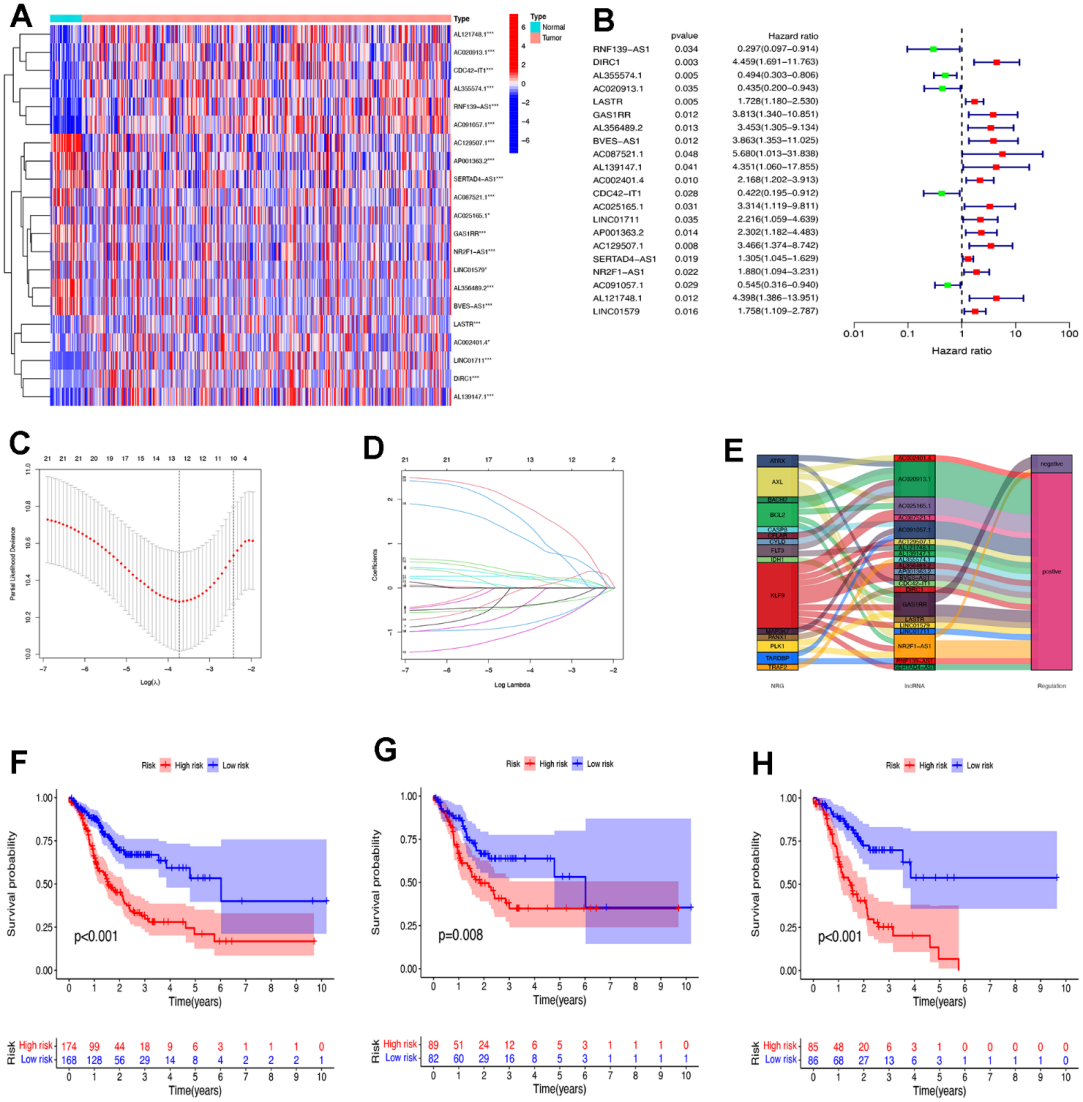
**Construct the risk prognostic model.** (**A**) The heat maps of expression profiles of 21 prognostic lncRNAs. (**B**) The forest maps of expression profiles of 21 prognostic lncRNAs by univariate Cox regression analysis. (**C**) The LASSO coefficient profile of 21 necroptosis-related lncRNAs. (**D**) The 10-fold cross-validation for variable selection in the LASSO model. (**E**) The Sankey diagram of 21 prognostic lncRNAs and necroptosis genes. (**F**) The survival analysis of all patients. (**G**) The survival analysis of patients in the test group. (**H**) The survival analysis of patients in the train group. NRG, necroptosis gene. * P < 0.05; ** P < 0.01; *** P < 0.001.

Based on the expression of lncRNAs, we constructed a patient risk model, namely the train group and the test group. Univariate Cox regression analysis identified 21 lncRNAs associated with necroptosis that had a significant OS correlation (P<0.05). The results were presented in forest maps and heat maps (Figure 3A, 3B). LASSO regression was performed on two groups of patients, and 9 lncRNAs associated with necroptosis in STAD were obtained after selecting the lambda (λ) values with the smallest cross-validation error (Figure 3C, 3D). The Sankey diagram illustrates that 19 lncRNAs had a positive correlation with necroptosis, while 2 had a negative correlation (Figure 3E). Based on the median risk score (Supplementary Figure 1), patients were categorized into high-risk and low-risk groups. The survival curve demonstrates that low-risk patients have a better prognosis (Figure 3F–3H).

### Assessment of the risk prognostic model associated with necroptosis-lncRNAs

**Figure 4:**
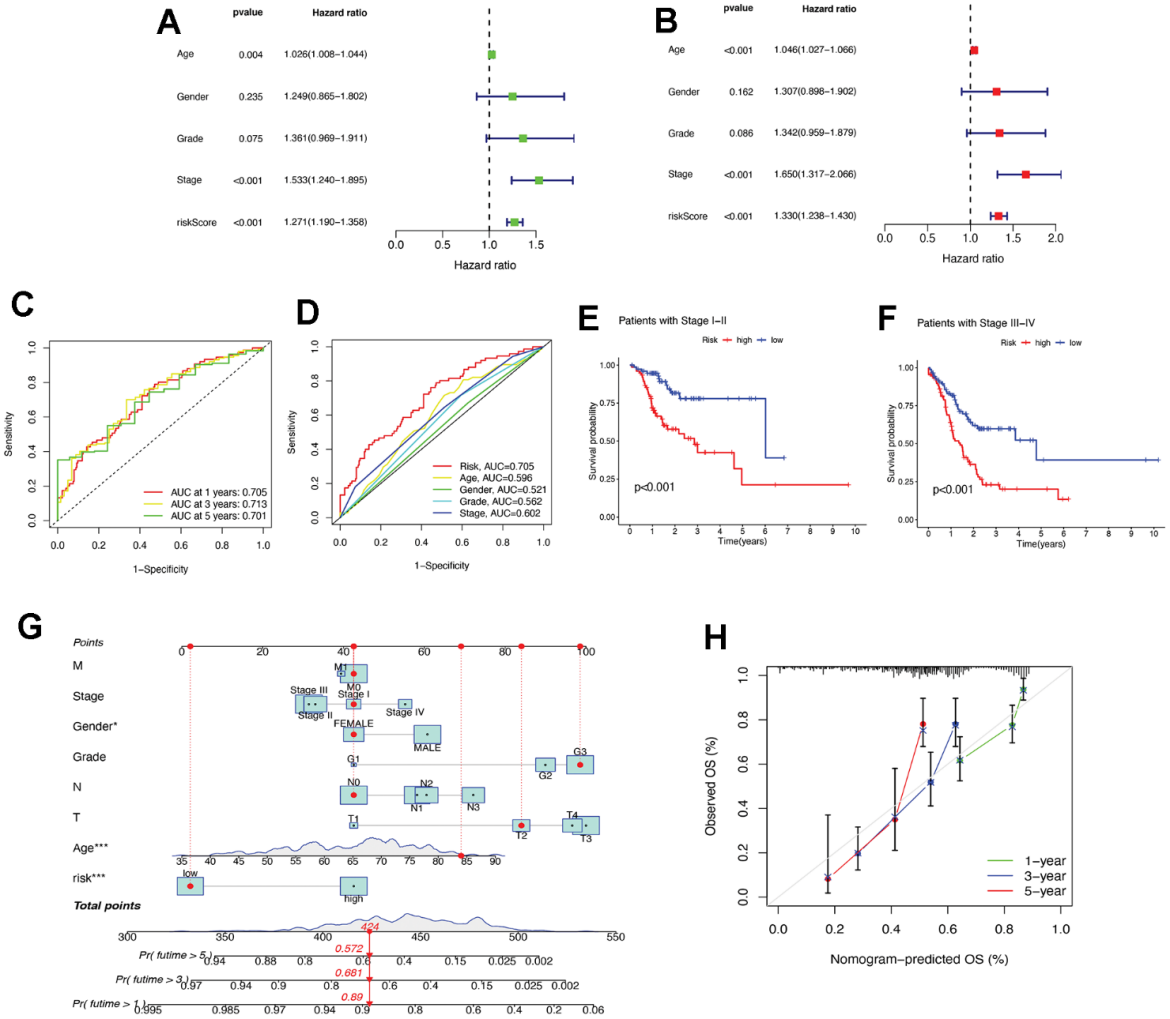
**Assessment of the risk prognostic model associated with necroptosis-lncRNAs.** (**A**) The uni-Cox analyses of clinical factors and risk score with OS. (**B**) The multi-Cox analyses of clinical factors and risk score with OS. (**C**) The 1-, 3-, and 5-year ROC curves. (**D**) The ROC curves of prognosis-related parameters. (**E**) The survival analysis of patients in the early stages (stages I, II). (**F**) The survival analysis of patients in the advanced stages (stage III, IV). (**G**) The nomogram of age, gender, risk score, and TNM staging. (**H**) The calibration curves for 1-, 3-, and 5-year OS. ROC, receiver operating characteristic; AUC, area under the curve; T, tumor; N, node; M, metastasis; OS, overall survival. * P < 0.05; ** P < 0.01; *** P < 0.001.

Univariate and multifactorial Cox regression analyses were performed on necrotizing necroptosis-related lncRNAs and OS, based on patient clinical characteristics and risk levels. The results showed that age, stage, and risk score were identified as independent prognostic parameters (Figure 4A, 4B). These findings suggest that the constructed riseScore could be considered a reliable prognostic model for STAD patients. The study analysed the sensitivity and specificity of the prognostic model in predicting patient survival time and prognosis-related parameters using the ROC curve (Figure 4C, 4D). The results showed that the constructed model had a high accuracy in predicting patient survival at 1, 3, and 5 years, with AUC values greater than 0.7. The risk score of the patient’s prognosis-related parameters had the maximum AUC value. According to the study, the risk score appears to be a more precise predictor of patient prognosis than other clinical features. The patients were categorized into early stages (I and II) and advanced stages (III and IV) based on their pathological stage. The model validation for clinical groupings indicated that risk scores could be utilized for STAD patients with varying tumor stages (refer to Figure 4E, 4F). A nomogram was constructed based on independent prognostic factors, including age, gender, risk score, and TNM staging. The patient in question, who is female, has stage I, T2N0M0, G3, and low-risk. The nomogram predicted a score of 424, indicating a probability of survival greater than 1 year of 0.89 (Figure 4G). The calibration curve demonstrates the high accuracy of the nomogram in predicting patient survival time (Figure 4H).

### Immune-related analysis of risk score of necroptosis-related lncRNAs

**Figure 5:**
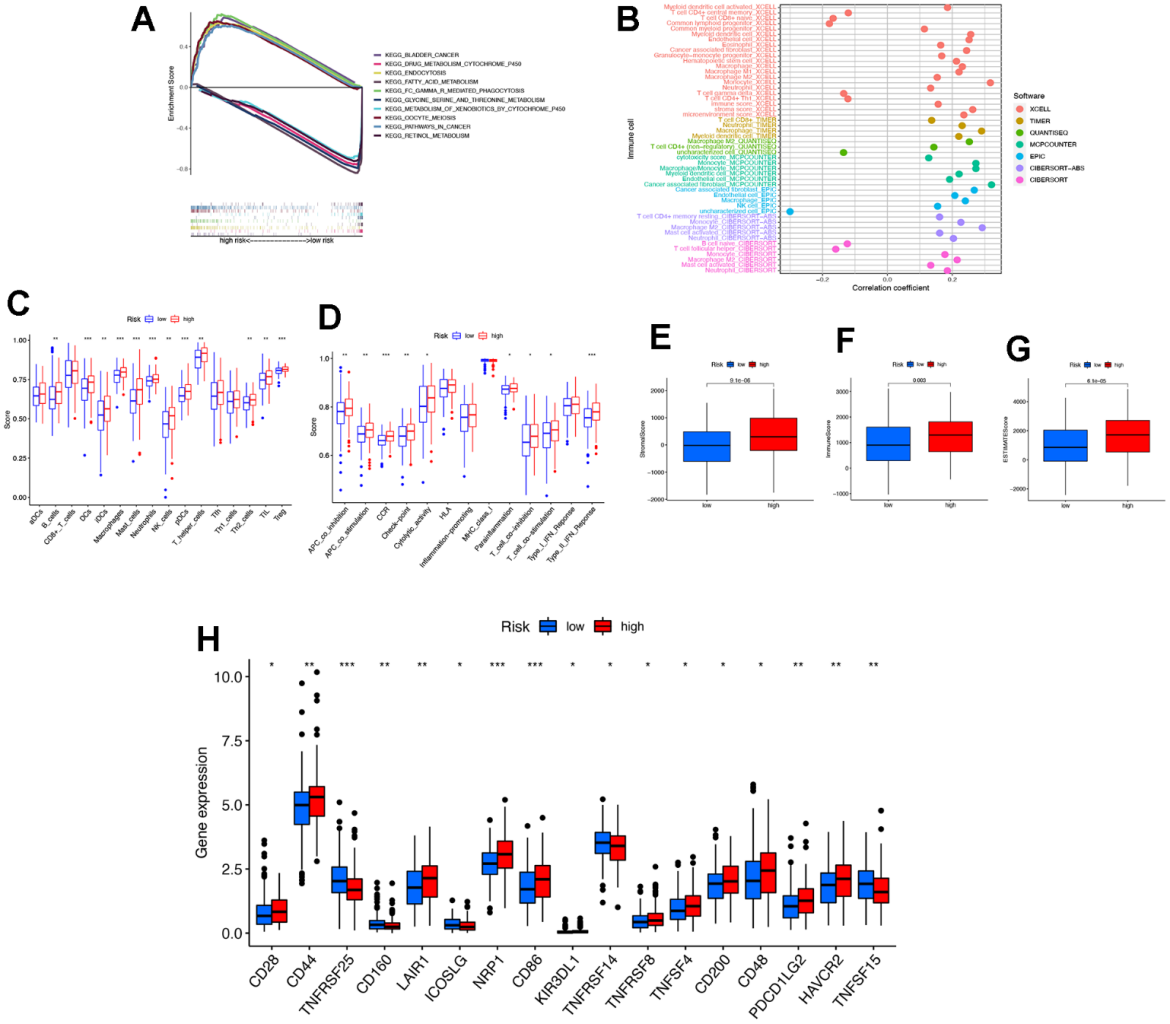
**Immune-related analysis of risk score of necroptosis-related lncRNAs.** (**A**) The GSEA analysis of the top 10 pathways significantly enriched the high-risk group and the low-risk group. (**B**) The immune cell bubble of risk groups. (**C**) The ssGSEA analysis of immune cells in risk groups. (**D**) The ssGSEA analysis of immune-related functions in risk groups. (**E**–**G**) The comparison of immune-related scores in risk groups. (**H**) The difference of 17 checkpoints expression in risk groups. KEGG, Kyoto Encyclopedia of Genes and Genomes; GSEA, gene set enrichment analysis; ssGSEA, single sample GSEA; * P < 0.05; ** P < 0.01; *** P < 0.001.

The Gene Set Enrichment Analysis (GSEA) was utilized to examine the KEGG pathway in both the high-risk and low-risk groups, with the aim of identifying differences in biological function. As depicted in Figure 5A, five pathways were found to be associated with the high-risk group, while five pathways were associated with the low-risk group. It is worth noting that the majority of the enriched pathways were significantly correlated with tumour invasion and immunity. The bubble plots analyzed on different software platforms suggest a positive correlation between most immune cells and the high-risk group (Figure 5B). The single sample GSEA (ssGSEA) difference analysis revealed differences in most immune cells between the high-risk and low-risk groups, with higher immune infiltration in patients in the high-risk group (Figure 5C). Similar results were observed in immune-related functions (Figure 5D). Upon analysis of the TME differences, it was found that the StromalScore, ImmuneScore, and ESTIMATEScore were higher in the high-risk group compared to the low-risk group (please refer to Figure 5E–5G). The differential analysis of immune checkpoint inhibitors revealed that immune-related genes were expressed more in the high-risk group than in the low-risk group (please refer to Figure 5H). According to the study, the lncRNA-related risk prognostic model may be helpful in selecting appropriate immune checkpoint inhibitors for STAD patients, which could lead to improved therapeutic efficacy. Additionally, drug sensitivity analysis was conducted on a total of 25 drugs related to STAD treatment (p<0.005, Supplementary Figure 2).

### The role of tumor clusters of necroptosis-related lncRNAs in immunotherapy

**Figure 6:**
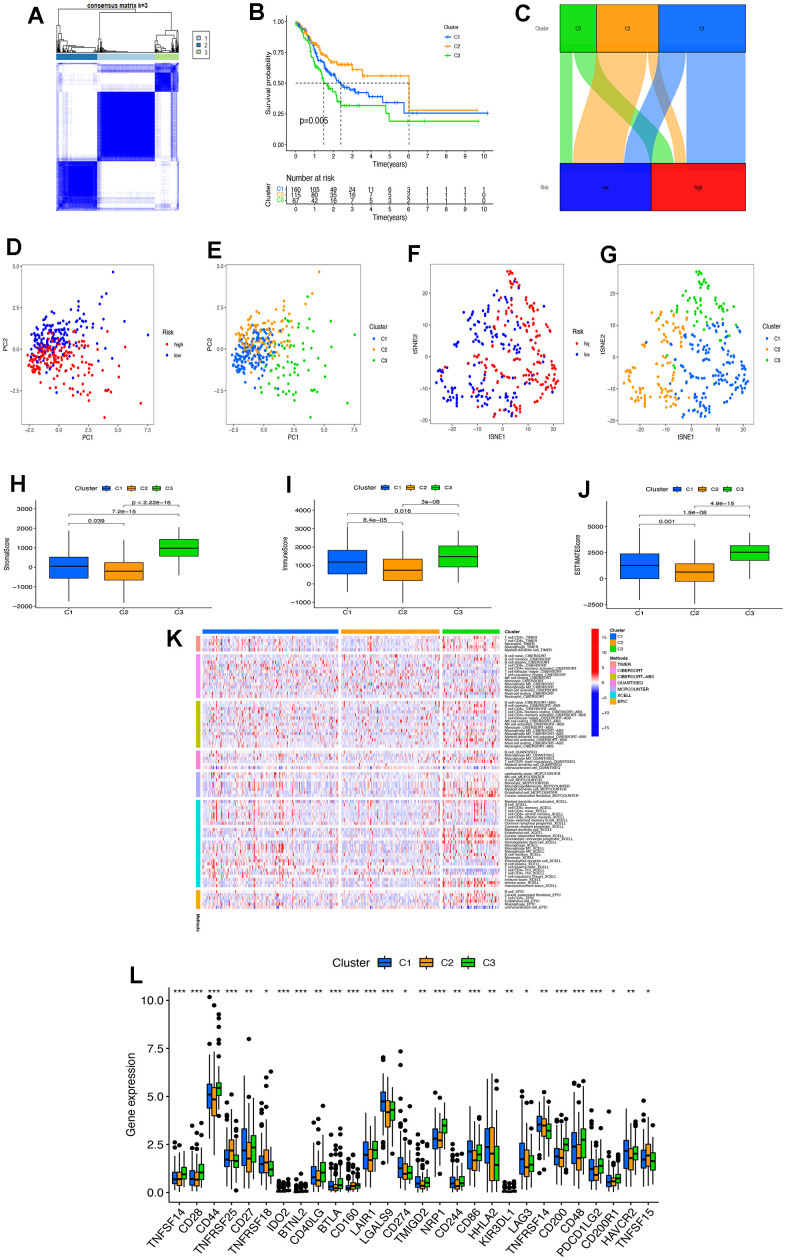
**The role of tumor clusters of necroptosis-related lncRNAs in immunotherapy.** (**A**) The necroptosis-related lncRNAs were divided into three subtypes according to the consensus clustering analysis. (**B**) The Kaplan–Meier analyzed the difference in the clusters. (**C**) The Sandel diagram of risk groups and the clusters. (**D**, **E**) The PCA of risk groups and clusters. (**F**, **G**) The t-SNE analysis of risk groups and clusters. (**H**–**J**) The comparison of immune-related scores in risk groups. (**K**) The heat map of immune cells with tumor clusters by different analytical methods. (**L**) The difference of 28 checkpoints expression in clusters. PC, principal component; tSNE, T-distributed stochastic neighbor embedding. * P < 0.05; ** P < 0.01; *** P < 0.001.

According to the consensus clustering analysis, the necroptosis-related lncRNAs were divided into three subtypes: cluster-C1 (C1), cluster-C2 (C2), and cluster-C3 (C3) (Figure 6A). Through K-M analysis, it was found that there was a difference in survival between the clusters, where C2 had an optimal survival time (Figure 6B). To explore the correspondence between STAD clusters and risk scores, we use the Sandel diagram for demonstration. The results showed that most of the C2 were low-risk patients (Figure 6C). Through PCA analysis, the results showed that patients in the high-risk and low-risk groups and different tumor clusters could be distinguished based on the expression of model lncRNAs (Figure 6D, 6E). The results of the t-SNE analysis also provided good validation of tumor clusters (Figure 6F, 6G). TME analysis showed differences between tumor clusters between StromalScore, ImmuneScore, and ESTIMATEScore (Figure 6H–6J). Among them, the C3 had the highest score in the analysis of the three TMEs. Figure 6K showed a heat map of immune cells with tumor clusters by different analytical methods. Through the differential analysis of immune checkpoints, it was found that almost all immune checkpoint-related genes were expressed differently in various tumor clusters (Figure 6L). Among them, CD44 expression was the highest. By comparing all drug sensitivities, we screened out 29 drugs with differences in drug sensitivity across different clusters (p<0.005, Supplementary Figure 3).

### Exploring the expression pattern of the identified necroptosis-related lncRNAs in the risk model

**Figure 7:**
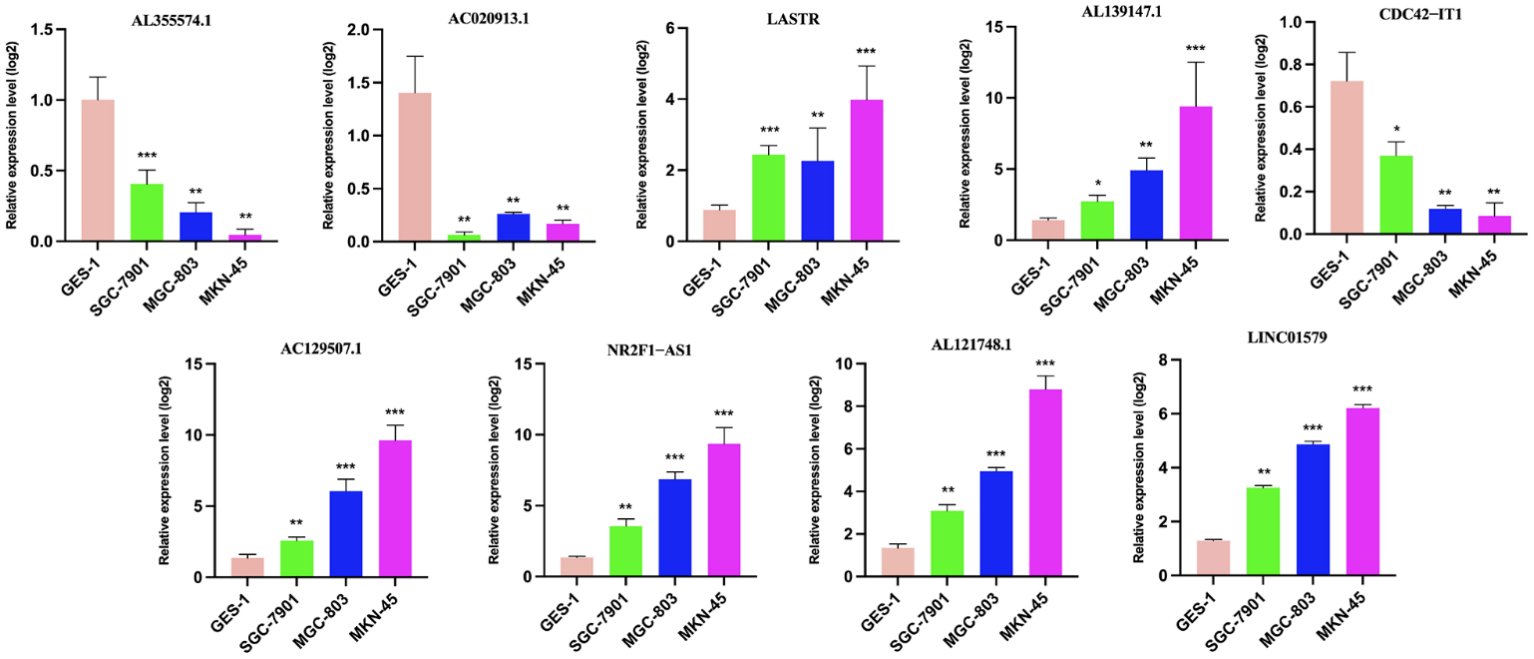
**The results of risk score gene expression identification.** qRT-PCR to evaluate the expression of necroptosis-related lncRNAs in GC cell lines and GES-1. GC, gastric cancer; qRT-PCR, quantitative real-time polymerase chain reaction. * P < 0.05; ** P < 0.01; *** P < 0.001.

To further validate the prognostic significance of the identified necroptosis-related lncRNAs in the risk model, we examined the expression of 9 lncRNAs in a panel of gastric cancer cell lines (SGC-7901, MGC-803, and MKN-45) and the human normal gastric epithelial cell line GES-1 by RT-PCR. The results showed that the expression of LASTR, AL139147.1, AC129507.1, NR2F1−AS1, AL121748.1 and LINC01579 was increased in gastric cancer cell lines relative to GES-1, and AL355574.1, AC020913.1, CDC42−IT1 was decreased in gastric cancer cell lines (Figure 7).

## DISCUSSION

Necroptosis is a mode of regulated cell death that has been recently discovered and is known to play a crucial role in maintaining a stable environment and embryonic development in the body. It has also been found to be a determinant of the pathological etiology of various human diseases [25]. However, the regulatory mechanism of necroptosis and its correlation with tumor pathological mechanisms are still areas that require further research. Additionally, it can remove tumor cells directly and release damage-associated molecular patterns (DAMPs) to recruit immune cells, creating a tumor microenvironment immune signaling system that can indirectly clear tumor cells [26]. Under different conditions, necroptosis can play a dual role in the development of tumorigenesis and anti-tumor therapy. It is important to acknowledge the potential benefits and drawbacks of necroptosis in the context of tumorigenesis and anti-tumor therapy. Further exploration of the key molecules of necroptosis and their molecular mechanisms of interaction with other genes may be necessary. Recent studies on long non-coding RNAs (lncRNAs) have shown their potential as important biomarkers for early diagnosis and as targets for STAD prevention. Therefore, it may be important to investigate the role of necroptosis-related lncRNAs in STAD and their impact on the anti-tumor immune response to potentially maximize the anti-tumor effect of necroptosis.

Necroptosis of tumor cells may promote tumorigenesis and metastasis by modulating the TME. In pancreatic ductal carcinoma, there have been observations of elevated expressions of RIPK1 and RIPK3. It was found that *in vivo* experiments involving the deletion of RIPK3 or inhibition of RIPK1 resulted in a delay in the progression of pancreatic ductal carcinoma in mice. An enhanced antitumor immune response was associated with this phenomenon, as evidenced by increased lymphocyte infiltration and decreased immunosuppressive medullary cell infiltration in RIPK3-deficient pancreatic ductal carcinoma [6]. A recent study used phosphorylated MLKL-specific antibodies to detect necrotizing apoptosis of tumor cells occurring in mouse models of breast cancer MMVT-PyMT [27]. According to these studies, it has been observed that tumor necroptosis took place *in vivo* and had a pro-tumor effect by stimulating the immune microenvironment, which in turn promoted tumor progression. It is also suggested that the necroptosis of non-tumor cells may contribute to the pro-tumor effects. For instance, necroptosis of intestinal epithelial cells has been reported to promote cancer by inducing colonic inflammation. Moreover, it has been observed that the use of necroptosis drug inhibitors, such as necrostatin-1 (Nec-1), in a dextran sulfate sodium-induced model of acute colitis significantly inhibits the occurrence of associated tumors due to colitis in mice [28]. Our study analysed the expression of 67 necroptosis-related genes in STAD and found evidence of necroptosis in STAD. Therefore, the induction of necroptosis in tumour cells could be considered as a promising therapeutic strategy for STAD treatment.

LncRNA HOTAIR, one of the earliest representative LncRNAs, has shown that the higher the expression level of HOTAIR, the greater the risk of STAD and the worse the prognosis. Li et al. demonstrated that BRD4 acts as a transcriptional regulator of MAGI2-AS3, promoting the epithelial-mesenchymal transition (EMT) of MAGI2-AS3, which in turn promotes the invasion and metastasis of STAD [29]. Several studies have confirmed that lncRNAs have been identified as reliable molecular markers for STAD-related tumors. LncRNAs have been found to be present in various bodily fluids, including peripheral plasma/blood, saliva, gastric juice, and urine. They have been shown to be stable and easy to detect. In a study conducted by Zhao et al, serum lncRNA HOTTIP was detected in 126 STAD patients and 120 healthy individuals (control group) [30]. After evaluating and comparing the diagnostic capacity of HOTTIP with other serum biomarkers, it was found that HOTTIP’s ability to diagnose STAD was significantly superior to that of carcinoembryonic antigen (CEA), CA19-9, and CA72-4, as indicated by its higher AUC. These findings suggest that HOTTIP and SPRY4-IT1 may be valuable diagnostic tools for STAD, and further research in this area is warranted. Additionally, Cao et al. (2018) reported that serum lncRNA SPRY4-IT1 was highly enriched in patients with STAD compared to normal individuals [31]. It has been observed that a high expression of SPRY4-IT1 is more commonly found in patients with large tumor volume, deep invasion depth, positive lymph node metastasis, and advanced gastric cancer. This observation suggests that SPRY4-IT1 can potentially serve as an early diagnostic marker and clinical staging indicator of STAD. In recent years, several studies have shown a close relationship between lncRNA and the prognosis of STAD patients, and its mechanism has gradually been confirmed. In their study, He et al. (2019) examined the expression of lncRNA UCA1 in 60 cases of STAD tissue and normal tissues. The results showed that the median survival of the low-expression group of UCA1 was significantly longer than that of the high-expression group of UCA1 [32]. This indicated that UCA1 overexpression predicts poor prognosis in patients with STAD. Chen et al. detected the expression levels of lncRNA VPS9D1-AS1 in 126 cases of STAD and normal tissues, and the results showed that their expression in tumor tissues was significantly downregulated [33]. The univariate and multifactor survival analysis showed that VPS9D1-AS1 expression was an independent prognostic indicator in patients with STAD. In our study, a total of 472 necroptotic-related lncRNAs were found. Through co-expression analysis, 21 lncRNAs associated with necroptosis have significant OS correlations. We believe that with the extensive and in-depth development of research, the biological effects and molecular mechanisms of necroptosis-related lncRNAs will be further elucidated, and provide new ideas and targets for the diagnosis and treatment of STAD.

In our study, a risk prognostic model consisting of 21 necroptosis-related lncRNAs was constructed to better assess the prognosis of STAD patients. Previous studies have demonstrated the crucial role of lncRNAs in regulating the immune response, gene activation, and immunophenotyping [34, 35]. Considering the promising results of immunotherapy in cancer treatment, immune-related lncRNAs have become a new area of interest. Necroptosis has been identified as a prognostic factor for various types of tumours, including pancreatic cancer [36], malignant glioma [37], and breast cancer [38]. As tumour prognosis is influenced by multiple factors, we combined other clinical features and constructed a nomogram to predict the individual survival rate of STAD patients. This approach provides a basis for clinical decision-making in STAD. Additionally, studies in tumour immunology have shown that necroptosis has anti-tumour effects. According to recent studies, it has been suggested that necroptosis could play a significant role in stimulating immunogenicity and promoting anti-tumor immune surveillance [39]. It has been observed that tumor cells undergo necroptosis after releasing interleukin-1α (IL-1α) to activate dendritic cells (DCs). The activated DCs, in turn, induce an anti-tumor immune response by producing cytotoxic IL-12 or activating CD8+ T cells to eliminate tumor cells [10, 11]. It has been observed that the release of DAMPs by tumor cells undergoing necroptosis has the potential to stimulate tumor antigen presentation in CD8+ T cells [12]. Additionally, RIPK3-mediated anti-tumor immune responses have been reported to involve natural killer T (NKT) cells. It was found that the deletion of RIPK3 impaired the antitumor activity of NKT cells [40]. In our study, we found that CD44 expression was the highest among all clusters. CD44 is a well-known marker of tumour stem cells and a key regulator of the EMT, which is involved in tumorigenesis, progression, and metastasis. The screening of immunotherapy targets and sensitivity to chemotherapy-related drugs was based on necroptosis-related lncRNAs. Hence, a better comprehension of the mechanism of necroptosis-related lncRNAs in STAD will contribute to a deeper understanding of how these lncRNAs boost the tumor immune response, which could be beneficial in investigating the mechanism of immunotherapy resistance in STAD.

## CONCLUSIONS

In conclusion, this study presents a comprehensive depiction of the tumour microenvironment landscape of necroptosis-related lncRNAs in STAD using a large amount of biological omics data from STAD patients. The findings were repeatedly verified in multiple dimensions, revealing the existence of three stable tumour clusters in STAD. These clusters are not only associated with the prognosis of patients with STAD but also significantly related to the patient’s subsequent treatment response and molecular typing. The quantitative evaluation of tumor cluster risk scores may enhance the precision of STAD immunotherapy, whether as a single drug or combination therapy. This study proposes a novel concept and foundation for necrotizing apoptosis-related lncRNAs to achieve more effective clinical translation and to accurately guide immunotherapy in STAD patients.

## Supplementary Material

Supplementary Figures

Supplementary Tables
