# Supplementary material for: The necroptosis-related signature and tumor microenvironment immune characteristics associated with clinical prognosis and drug sensitivity analysis in stomach adenocarcinoma
Source: Aging (Albany NY). 2024 Mar 27;16(7):6098–117. doi: 10.18632/aging.205690 (PMC11042952; doi:10.18632/aging.205690)
Supplement: Supplementary Figures [file aging-16-205690-s001.pdf]

SUPPLEMENTARY FIGURES

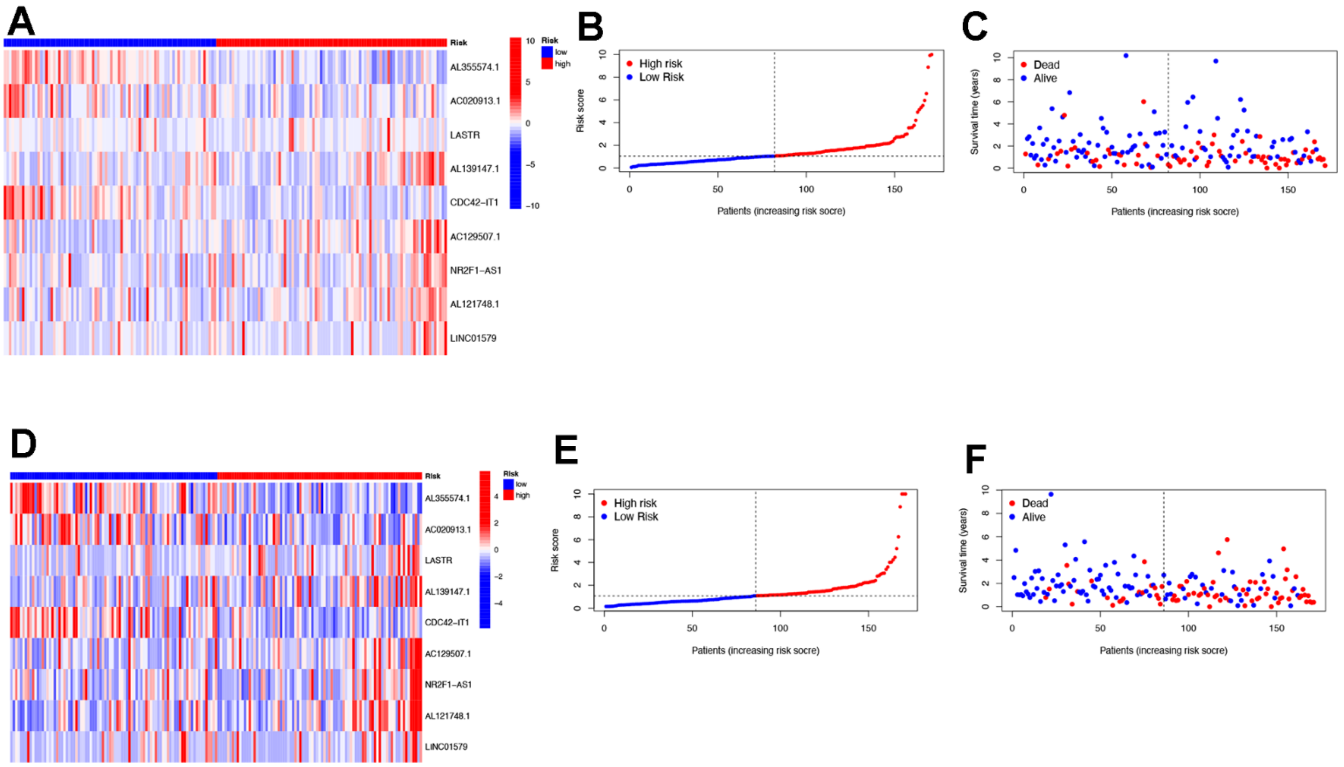

Supplementary Figure 1. (**A**) The heat map of 9 lncRNAs expression in the test. (**B**) Exhibition of necroptosis-related lncRNAs model based on risk score of the test. (**C**) Survival time and survival status between low- and high-risk groups in the test. (**D**) The heat map of 9 lncRNAs expression in the train. (**E**) Exhibition of necroptosis-related lncRNAs model based on risk score of the train. (**F**) Survival time and survival status between low- and high-risk groups in the train.

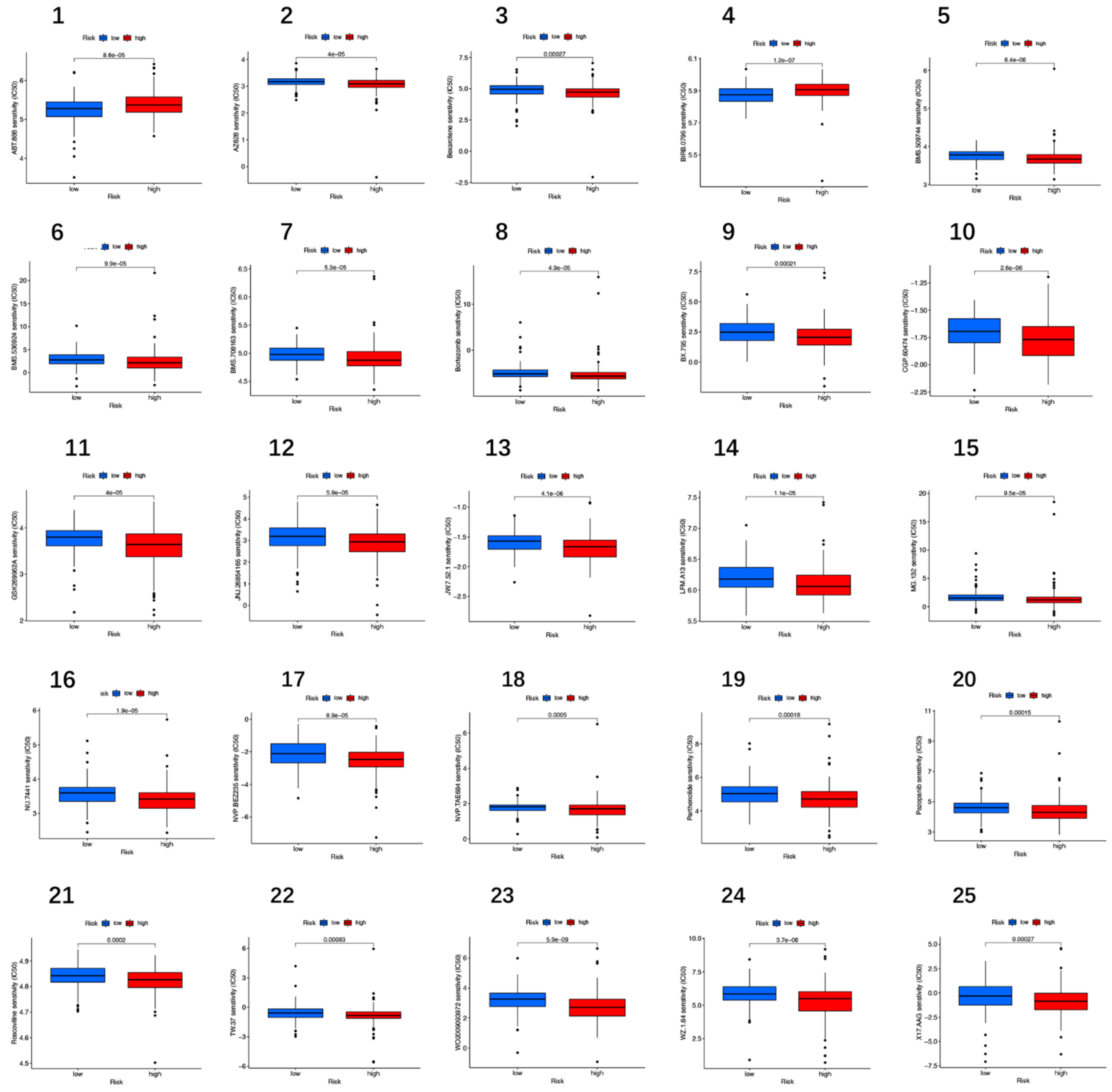

Supplementary Figure 2. (**1**–**25**) 25 immunotherapeutic drugs showed a significant IC_{50} difference in different risk groups (all $p<0.05$).

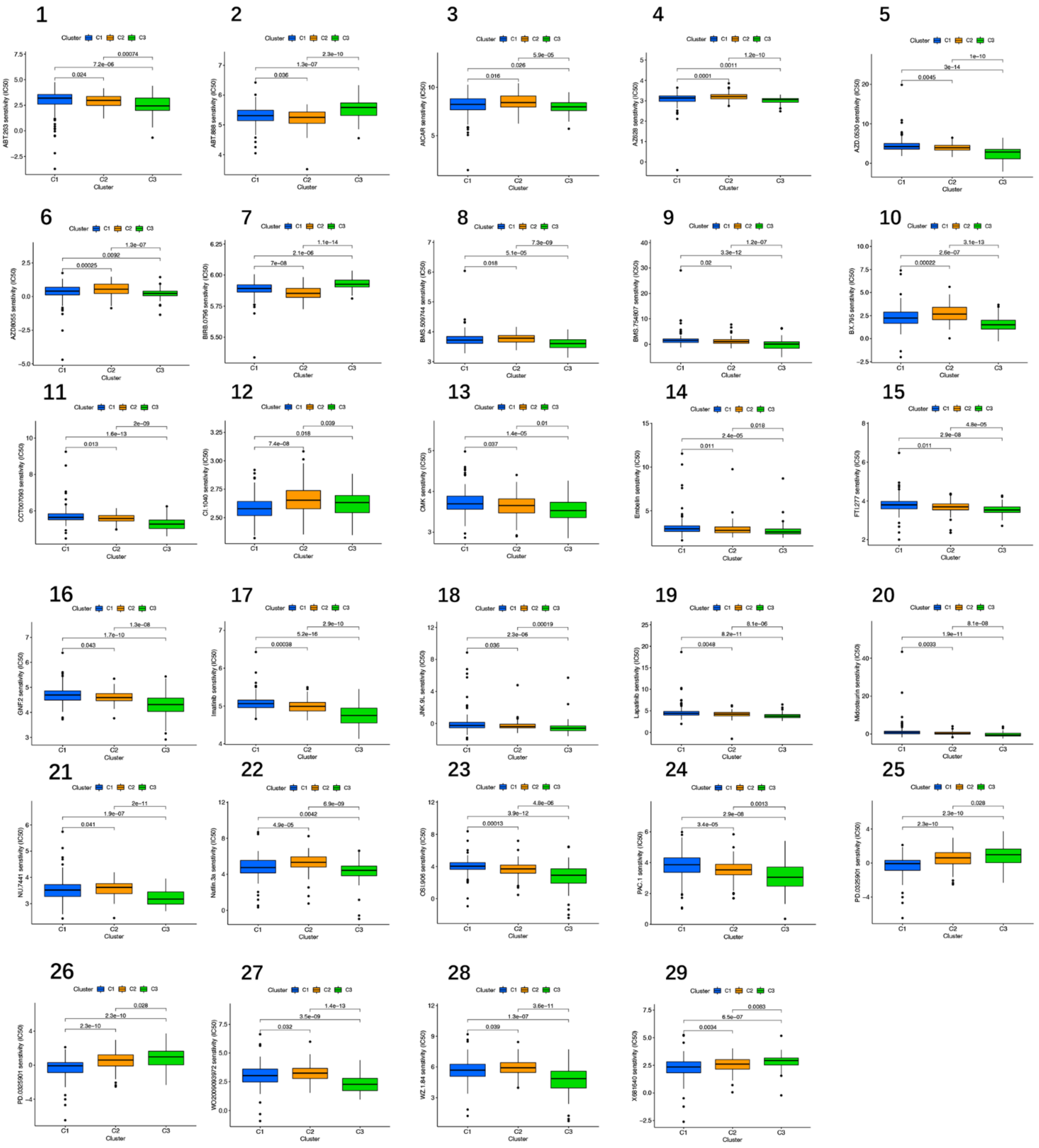

Supplementary Figure 3. (**1**–**29**) 29 immunotherapeutic drugs showed a significant IC_{50} difference in different clusters (all p<0.05).
