# Supplementary material for: The necroptosis-related signature and tumor microenvironment immune characteristics associated with clinical prognosis and drug sensitivity analysis in stomach adenocarcinoma
Source: Aging (Albany NY). 2024 Mar 27;16(7):6098–117. doi: 10.18632/aging.205690 (PMC11042952; doi:10.18632/aging.205690)
Supplement: Supplementary Tables [file aging-16-205690-s002.pdf]

SUPPLEMENTARY TABLES

Supplementary Table 1. The 67 necroptosis-related genes.

FADD	TRAF2	SIRT2	CD40	MYC	IPMK
FAS	PANX1	SIRT1	BCL2L11	BCL2	ID1
FASLG	OTULIN	PLK1	EGFR	HDAC9	IDH1
MLKL	CYLD	MPG	DDX58	SIRT3	TNFRSF1A
RIPK1	USP22	BACH2	TARDBP	HSPA4	MAPK8
RIPK3	MAP3K7	GATA3	APP	KLF9	AXL
TLR3	SQSTM1	MYCN	TNFRSF21	HSP90AA1	RNF31
TNF	STAT3	ALK	TNFRSF1B	ITPK1	LEF1
TSC1	DIABLO	ATRX	HAT1	CDKN2A	ZBP1
TRIM11	DNMT1	TERT	BNIP3	IDH2	BRAF
CASP8	CFLAR	SLC39A7	TNFSF10	STUB1	SPATA2
FLT3					

Supplementary Table 2. The primers used in the study.

Gene name	Forward primer (5'→3')	Reverse primer (5'→3')
AL355574.1	GGAGGGCAGAGAGCAACGTA	CGCCTCTACAGACAGCACTC
AC020913.1	GAATGGCAAAGCACTTGGGG	AGCTCTCCTTGCAGGTAGGT
LASTR	AGTGGGTGAAGTCCTGGTT	GGCTGAAGGGTTTAGATG
AL139147.1	CACAGCCAAAACCAAACTCCT	TGGGTTGCCATTCACTGACT
CDC42−IT1	GCCGGAGAACGAATGTGATG	TCTTTTTGTTTACCGGAGTTCCA
AC129507.1	GGACAGATGGTGGGAGAGGAA	ATGCTGACGAGGTTTGGAGAATC
NR2F1−AS1	GCCCATGATGAACCTGTTTT	TTACATCACGGCATGGTAGC
AL121748.1	AACAAGGCACAAAAGGGGAAAA	AGCAAACGCAAGGCGAAGG
LINC01579	TCCCAGTGAAGAGAGAGCGA	CTAAGTTCCACGTCACGGCT
GAPDH	AATGGGCAGCCGTTAGGAAA	GCCCAATACGACCAAATCAGAG
